# A Comparative Evaluation of the Effects of Manufacturing Parameters on Mechanical Properties of Additively Manufactured PA and CF-Reinforced PA Materials

**DOI:** 10.3390/polym15010038

**Published:** 2022-12-22

**Authors:** Mumin Tutar

**Affiliations:** Department of Technology Sciences, Air NCO Higher Vocational School, Turkish National Defence University, İzmir 35415, Türkiye; mtutar@msu.edu.tr; Tel.: +90-232-251-16-00

**Keywords:** additive manufacturing, fiber reinforcement, mechanical properties

## Abstract

Nowadays, 3D printers, which have a wide range of applications, continue to become widespread and are more and more common. As a result, in addition to the visuality of the parts produced with this method, their mechanical properties have gained importance depending on where they are used. In addition to the many conveniences, it provides during the design and production phases according to traditional methods the features of the printing parameters used, especially the printing direction and angle, which vary depending on the direction. For this reason, it is necessary to determine how the mechanical properties change depending on these parameters. In this study, compression, tensile, and bending tests were carried out with samples produced by the FDM method using polyamide (PA) and carbon fiber reinforced PA (PA-CF) filaments. The effects of fiber reinforcement, raster angle, and frame on the mechanical properties were evaluated. The porosity of manufactured parts was also discussed.

## 1. Introduction

The concept of a 3D printer, which was introduced as an idea in the early 1980s, has become an important pillar of today’s developing technology. It has made the desired final product faster and prototyping and rapid production possible by leaving behind many intermediate processes and intermediary persons. In particular, the FDM (fused deposition modeling) method, in which polymer materials are used and needs-oriented designs are printed quickly, is used for this purpose significantly.

The name 3D printing is given to the formation process of the three-dimensional model, which is mainly formed by the overlapping of two-dimensional layers. While traditional production methods obtain a part by material subtraction, 3D printers construct the part layer by layer, with no or insignificant amount of material loss [1,2].

The key advantage of 3D printing over traditional methods is that there are virtually no limits to geometry complexity. Thus, it is possible to produce parts that are difficult or impossible to produce with traditional methods at a lower cost. However, in cases where mass production is required, high cost per piece and high production time limit the use.

Another important point is whether the parts produced for functional purposes will provide the desired mechanical strength in the place where they will be used. Currently, the FDM technique is mostly used for production with polymer material, although metal printing is also possible. The final mechanical properties of the polymer part produced with FDM are significantly affected by the printing parameters, as well as depending on the base polymer used [3,4,5,6].

In areas where mechanical properties are important, the use of fiber-reinforced filaments has found application. The 3D-printed reinforced polymer applications are increasing rapidly due to improvements in mechanical properties, as well as the number of publications in this field [7,8,9].

The 3D printing of fiber-reinforced polymers is classified into two main groups: short/chopped and continuous fiber printing. Chopped reinforced filaments could be custom-made with an intended percentage of fibers (carbon, glass, or Kevlar) or there are also commercial pre-impregnated filaments. Low density, low thermal expansion, and thermal conductivity make CF stand out compared to other reinforcements [7,9,10].

If printing will be conducted with conventional FDM printers, hardened nozzles are needed due to the abrasive nature of the fibers. Dedicated commercial printers, such as Markforged, use Onyx (commercial chopped CF-reinforced nylon/PA) and are further reinforced with continuous CF. A list of printers and types of reinforced filaments that could be printed with them is given in [7,8].

PA is a polymer known for its superior mechanical properties, i.e., tensile strength, flexibility, and impact resistance, as well as chemical and corrosion resistance. Having these improved properties, it is used not only in prototyping but also in functional parts in areas such as machines, electronics, and the food industry. Reinforced PA has also found application in the automotive and aerospace industries [7].

When the literature is reviewed, it was seen that most of the studies on CF-reinforced PA are carried out by dedicated printers, such as Markforged [7,8]. The mechanical properties of chopped CF-reinforced PA printed with conventional FDM printers have not been studied sufficiently and have been the focus of only a few studies [11,12,13]. Moreover, the studies on the mechanical property of FDM printed parts focus on tensile and flexural properties, and it is seen that the compression properties are also less studied [8,12,14]. Thus, the motivation of this study was to determine the mechanical properties of a commercially available chopped CF-reinforced PA and compare it with the unreinforced PA. For this purpose, compression, tension, and bending tests were carried out.

## 2. Materials and Methods

Filaments used in this study are Ultrafuse PA (a polyamide) and Ultrafuse PAHT CF15 (a high-temperature polyamide-based filament filled with 15% chopped carbon fibers) produced by BASF (Ludwigshafen, Germany). For simplicity, PAHT CF15 is referred to as PA–CF in the remainder of the study. A modified Creality Ender 3 Pro (Shenzhen, China) branded 3D printing machine was used for fabrication of the samples. Printing parameters used in the FDM machine are presented in Table 1. The infill percentage was 100% for all samples. Due to the abrasive nature of carbon fiber-reinforced filaments, a hardened steel nozzle was used, as well as a glue stick for better bed adhesion. After printing, the samples were stored in Tupperware airtight containers (Grafton, MA, USA) containing silica gel until testing.

ASTM D638, ASTM D695, and ASTM D790 were followed for tensile, compression, and 3-point bending tests, respectively. Tensile test specimens were printed as Type 1 with a 4 mm thickness. They were repeated five times and compression tests were performed three times since they were more repetitive. All tests were carried out at room temperature with a WDW 100 electronic universal testing machine (Jinan, China). Table 2 presents the standards used and other test information, i.e., printed specimen dimensions, test speed, and the number of specimens tested.

Tensile test specimens of PA were printed as unframed with various infill raster orientations of 0°, ±45°, and 90° and PA–CF, both as framed and unframed, having raster orientations of 0°, 45°, ±45°, and 90°.

**Figure 1:**
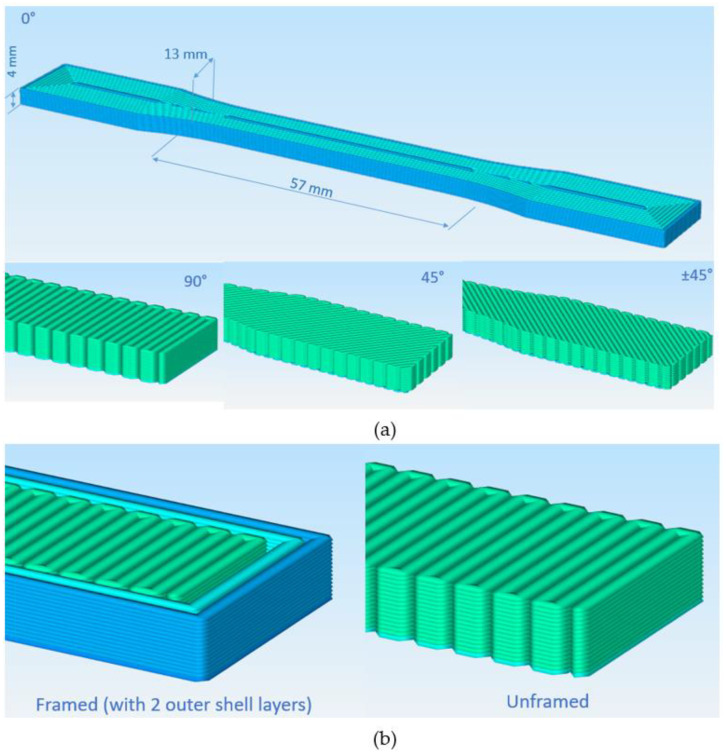
ASTM D638 tensile test specimens (**a**) according to infill raster angles and (**b**) frame configuration.

**Figure 2:**
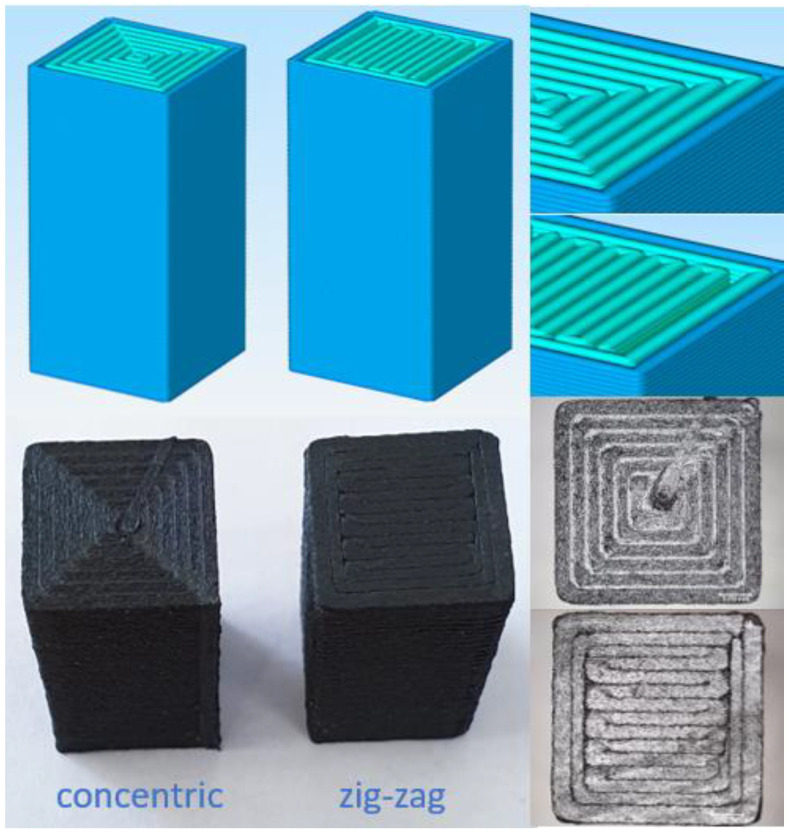
Sliced and real images showing print patterns of compression test specimens.

Framed configurations comprised two shell layers outside and were used to investigate the effect of the outer shell on tensile properties. In Figure 1, sliced images of different configurations are demonstrated. Compression test specimens were also printed in two different patterns: concentric and zig-zag (Figure 2). The printing pattern of bending test specimens was similar to tensile specimens, comprising different raster orientations, without any frame. A nomenclature to represent specimens with different filament material, raster orientation, and frame info was constituted and given in Table 3.

The mass of printed specimens was measured by a Sartorius BP2215 analytical lab scale (Darmstadt, Germany) with a precision of 0.1 mg for porosity calculations.

## 3. Results and Discussion

### 3.1. Tensile Tests

#### 3.1.1. The Effect of Fiber Reinforcement

**Figure 3:**
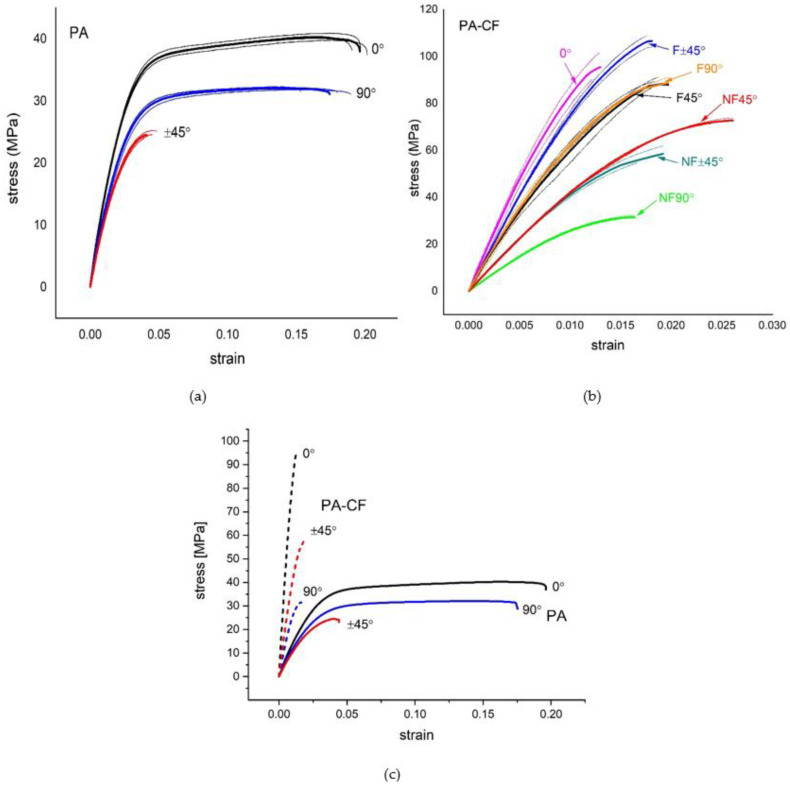
Engineering stress–strain curves obtained by tensile tests. (**a**) PA, (**b**) PA–CF (F: framed, NF: not framed) (thin curves are individual tests whereas bold ones are the averaged curves), and (**c**) a comparison of the averaged curves for PA and PA–CF (solid curves are PA and dashed curves are PA–CF).

Engineering stress–strain curves for all tests of PA and PA–CF tensile samples are illustrated in Figure 3. Figure 3a,b include the repeated test curves with the average curves for each configuration to display the consistency of the results. In addition, the calculated averaged tensile properties according to these curves are listed in Table 4.

It should be recalled that PA test samples were printed with unframed configuration only; therefore, in Figure 3c, where PA and PA–CF are compared, unframed PA–CF tensile curves are included. The effects of reinforcement regardless of the raster angle can be generalized as follows:

- A decrease in toughness and ductility, and
- An increase in tensile strength and stiffness.

The obtained results are in line with the literature [7,8,15].

#### 3.1.2. The Effect of the Raster Angle

**Figure 4:**
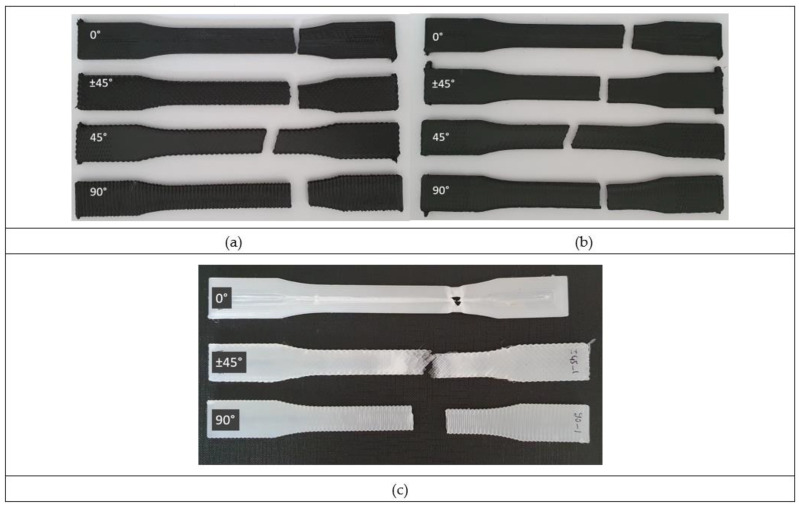
Tensile test fracture patterns. (**a**) PA–CF without a frame (**b**). PA–CF with a frame. (**c**) PA without a frame.

As seen in Figure 3c, PA shows a consistent decrease in ultimate strength with raster angles of 0°, 90°, and ±45°, respectively. One of the most remarkable points here is the elongation at the break value of the ±45 raster angle is much lower compared to the other two raster angles. The reason for this is a failure due to the delamination between different layers being more effective at this raster angle (Figure 4c). For PA using the ±45° raster angle instead of 0°, there is a 39% decrease in strength with a 79% loss in ductility. In terms of elastic modulus values, the 0° angle was stiffer, while the difference between the 90° and ±45° angles was not significant.

Continuing with Figure 3c, for unframed PA–CF samples, the 0° raster angle exhibits the highest strength again, yet the ±45° raster angle has higher strength than the 90° raster angle in this case. This is simply because delamination of layers is not observed for PA–CF. Instead, the failure takes place due to fracture between adjacent rasters in the case of 90° (Figure 4a). The ±45° raster angle strength depends on both bonding strength and filament strength. The fiber reinforcement in the case of the ±45° raster angle improves interfacial adhesion between layers and thus, the strength of PA was improved by 137%. The fiber reinforcement in the case of the 0° raster angle is again very effective because this time the fibers are oriented along the loading. The fiber reinforcement in the case of the 90° raster angle was not effective because failure takes place due to the weak bonding strength between adjacent rasters.

#### 3.1.3. The Effect of a Frame

There are few studies that investigated the effect of shell layers on mechanical properties. All these publications, except one of them, focused on the effect of the number of shell layers. The authors reported that increasing the number of shells has improved the mechanical behavior [16,17,18,19,20].

In this study, two shell layers were added to PA–CF ± 45, PA–CF45, and PA–CF90 samples to determine the effect of shell layer addition, and the “f” character was used at the end of the abbreviated name of the samples to distinguish the framed samples. Both Figure 3b and Table 4 can be referred to for comparison.

**Figure 5:**
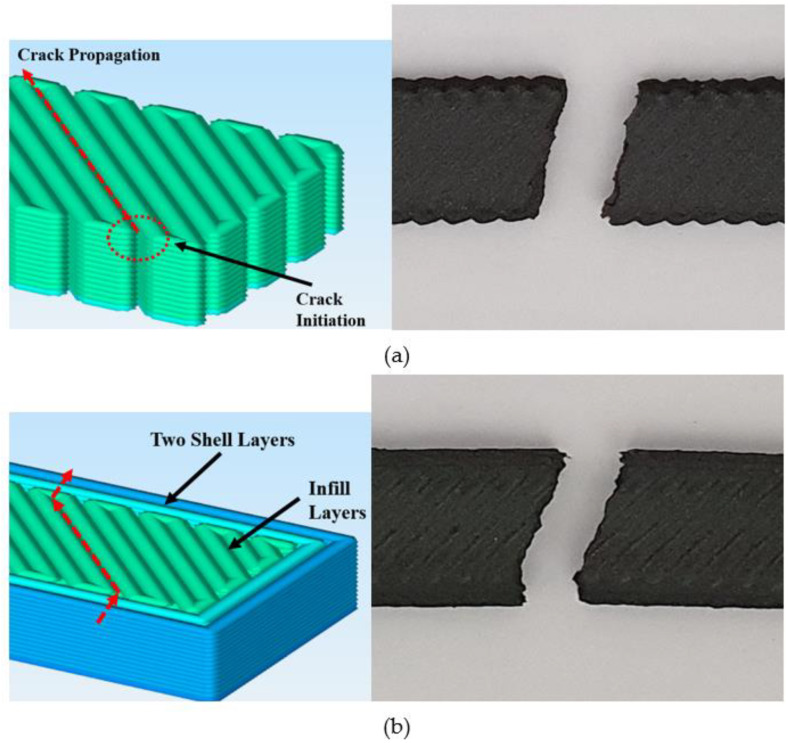
Crack initiation and propagation in the 45° raster angled of the framed and non-framed samples. (**a**) Non-framed. (**b**) Framed.

The shell layers produced along the loading direction showed an effect to decrease the surface roughness (Figure 5). Thus, the discontinuities (grooves) of the outer surface in the absence of shell layers create stress intensity and earlier crack formation. This is the reason that the tensile strength of framed samples is higher than the unframed ones, as seen in Figure 3b.

The addition of a frame increases tensile strength by 183%, 83%, and 21% for raster angles 90°, ±45°, and 45°, respectively. In the case of the 90° raster angle, for which the tensile strength relies on the strength of the bonding between rasters, the strength increase was more pronounced than the others.

Figure 5 illustrates the sliced images of the samples with (PA–CF45f) and without (PA–CF45) a frame. The schematic and the actual fracture images display how the frame affects the crack propagation. Without frame configuration, the crack starts from the surface discontinuity and propagates with the direction parallel to the raster orientation (45°). With the frame, the crack starting from the edge firstly propagates perpendicular to the loading direction in the framing part and changes its direction to parallel to raster orientation until it comes to the frame on the opposite side.

**Figure 6:**
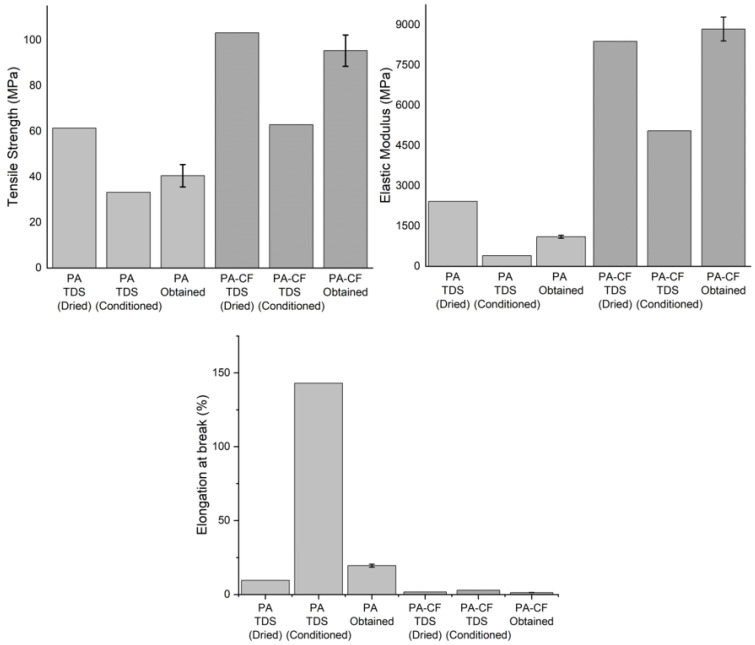
Comparison of mechanical properties obtained by this study and the TDS (the 0° raster angle).

The comparison of mechanical properties obtained from the tensile test and the technical data sheet (TDS) of the used filaments is given in Figure 6 [21,22]. The properties are given for “dry” and “conditioned” states in the TDSs. To ensure printability, the recommendation for drying is 4 to 16 h in a hot air dryer at 70 °C, and for the best mechanical properties, the optimum drying recommendation is at least 40 h at 80 °C in a vacuum oven. It is well known that polymers, especially polyamide, are very sensitive to moisture which changes their mechanical properties. In this study, to keep samples with minimum humidity as possible, produced samples were kept in an airtight storage box until the test. In addition, silica gel desiccant with a color indicator was used. Because the adsorption capacity of silica gel is related to the temperature and relative humidity of the air, it does not create a drying effect, as mentioned in the TDS. It is also noted in the TDS that, to ensure constant material properties, the material should always be kept dry. For these reasons, the obtained mechanical properties from the tests are found to be between the dry and conditioned states given in the TDS.

For the PA polymer, while increasing moisture decreases the tensile strength and stiffness, it increases the elongation at break. These results are in accordance with previous studies. Additionally, it can be noted that the obtained tensile strength values of the PA were closer to the conditioned state, while the tensile strength of PA–CF was closer to the dried state. This result also showed that the addition of fiber reduced the rate of change of mechanical properties due to moisture [23,24,25].

Most researchers reported that interfacial adhesion between fiber and a matrix had a crucial role in the mechanical properties of fiber-reinforced thermoplastics. In low humidity conditions, as in this study, the dominant determinant is fiber reinforcement, which is also reflected in the results obtained. While the tensile strength value of PACF0 is between dry and conditioned values, elongation at break values is lower than dry values. This difference is attributed to possible variations due to process parameters and production environments [23,24,25,26].

This section may be divided into subheadings. It should provide a concise and precise description of the experimental results, their interpretation, as well as the experimental conclusions that can be drawn.

### 3.2. Compression Tests

**Figure 7:**
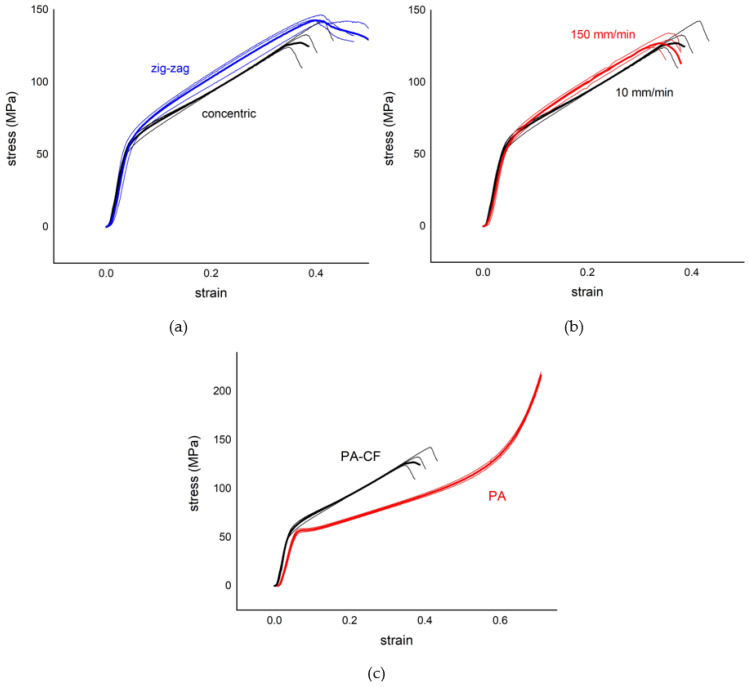
Engineering stress–strain curves obtained by the compression tests of PA–CF effect on (**a**) the printing pattern, (**b**) the compression loading rate, and (**c**) the comparison of PA–CF and PA (thin curves are individual tests whereas bold ones are the averaged curves).

**Figure 8:**
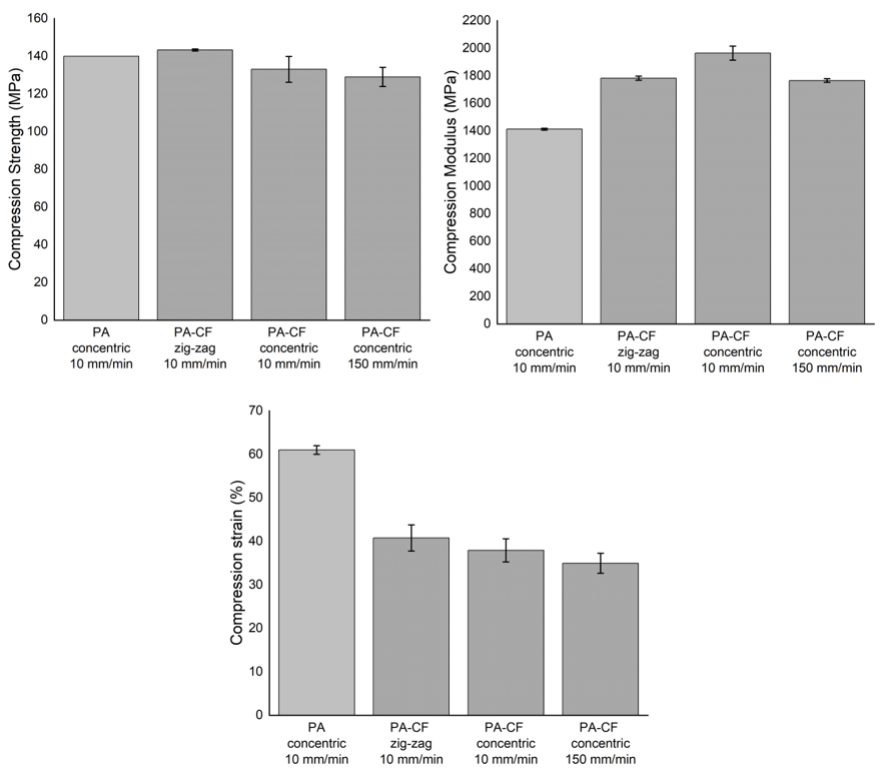
Compression properties: compression strength, compression modulus, and compression strain.

Stress–strain curves obtained by compression tests for the PA and PA–CF are presented in Figure 7. PA–CF was tested for two different compression rates (10 and 150 mm/min) and printing patterns, as given in Figure 2: concentric and zig-zag. All samples were 18 mm compressed which equals a 0.7 strain. The calculated mechanical properties from the compression tests were given in Figure 8 for comparison.

**Figure 9:**
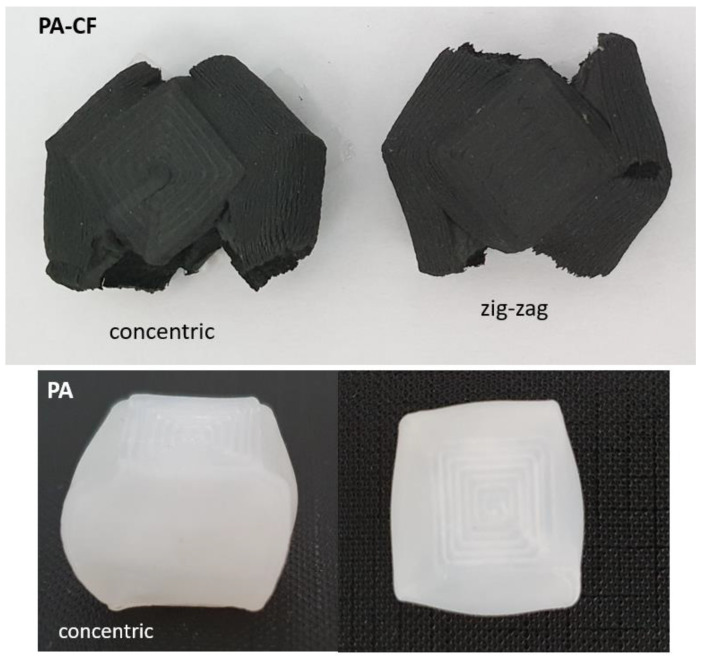
Tested compression samples.

The PA sample shows a compressive behavior similar to a cellular structure due to the inherent voided structure of the FDM printed sample. The compressibility continues until a densification strain where the structure starts behaving more rigid and the stiffness rises abruptly. Since no failure was observed in these samples (see Figure 9) the compressive strength and strain values are taken from this densification point, which is calculated by using the tangents method graphically. PA–CF, which has a brittle structure, behaves more stiffly after the elastic region where the two materials show a similar stiffness.

When applied different compression rates, there was not an apparent rate sensitivity in the range between 6.6 × 10^−3^/s and 98.4 × 10^−3^/s. The same conclusion can be made for the printing patterns: the effect of printing patterns on compression mechanical properties has been minimal.

### 3.3. Three-Point Bending Tests

**Figure 10:**
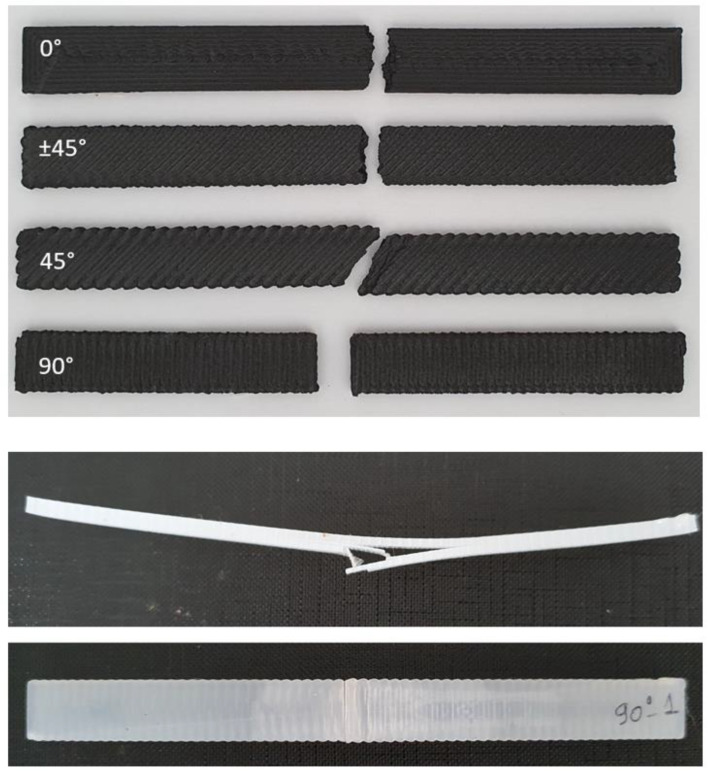
Failed samples of 3-point bending tests.

Flexural stress–strain curves of the 3-point bending tests are given in Figure 10. The higher flexural strength was observed for the 0° raster angle for both PA and PA–CF. Similar to tensile samples, this clear difference is attributed to the higher mechanical strength of the filaments compared to the bonding strength. The fracture path was along the weak interface between adjacent rasters for the 90° and 45° raster angles, whereas it proceeded perpendicular to the loading direction (Figure 10). Due to the changes in the fracture path in the PA–CF layer at ±45°, a higher strain was observed.

PA samples showed no fracture, except the 90° raster angle, which failed due to the weak interface between the layers resulting in the delamination of the adjacent layers (Figure 10).

### 3.4. Porosity

Porosity is an important factor that affects the mechanical performance of 3D-printed parts. Unlike conventionally manufactured parts, additionally manufactured parts have an inherent voided structure. The filaments without fiber have inter-bead voids that are formed between the adjacent beads and layers. Fiber-reinforced parts, however, have porosity also inside the bead which is called intra-bead voids.

Tekinalp et al. showed that with the increase in carbon fiber content to ABS, the inter-bead porosity decreases and intra-bead porosity increases. They pointed out that fiber addition creates smaller inter-bead gaps due to the reduced die swell effect and the increase in thermal conductivity resulting in smaller beads and, therefore, smaller inter-bead gaps. In their study, void volume fraction was identified between 16% and 27% for the fiber content between 0% and 40% [27].

Similarly, Ning et al. observed an increase in porosity with the increasing amount of carbon fiber while the porosity decreased from 9.04% to 3.27% when the carbon fiber level increased from 10% to 15% [28].

In this study, the porosity values for both filaments were calculated with the following formulas to compare the reinforced and unreinforced filament porosities [28].

(1) $$P=\frac{V_{t}-V_{a}}{V_{t}}$$

Here, *V_t_* is the theoretical volume of the specimen including the pores and *V_a_* is the actual volume of the specimen without the pores. As for the porosity calculated above, it should be noted that the volume of the designed object in CAD could differ from the actual one due to the shrinkage of the FDM printed parts. Thus, here, the latest volume (*V_t_*) of the printed compression specimens is measured using a Mitutoyo caliper. For ease of measurement, compression specimens were used. *V_a_* is actual volume, meaning the volume with the porosity is calculated by Equation (2):

(2) $$V_{a}=\frac{m\times wt{\%}_{PA}}{\rho_{PA}}+\frac{m\times wt{\%}_{CF}}{\rho_{CF}}$$

where *m* is the actual mass of the specimen, *ρ_PA_* is the density of *PA* (1.083 g/cm^3^), and *ρ_CF_* is the density of carbon fiber (1.55 g/cm^3^). The density of *PA* specified in the TDS is 1.115 and 1.05 g/cm^3^ for dry and conditioned states, respectively. Since the values in this study are between the dry and conditioned states, the density for *PA* is calculated as the average of these values. As for the carbon fiber density, an average value of 1.8 g/cm^3^ is used, as stated in [29]. The actual dimensions of the specimens were measured by a Mitutoyo caliper and the mass of the specimens are weighed by a Sartorius precision scale (readability of 0.0001 g). Finally, using Equations (1) and (2), the average porosities calculated for PA was 6.3% and PA–CF was 10.3%. So, the 15% carbon fiber addition increased the porosity by 4%.

**Figure 11:**
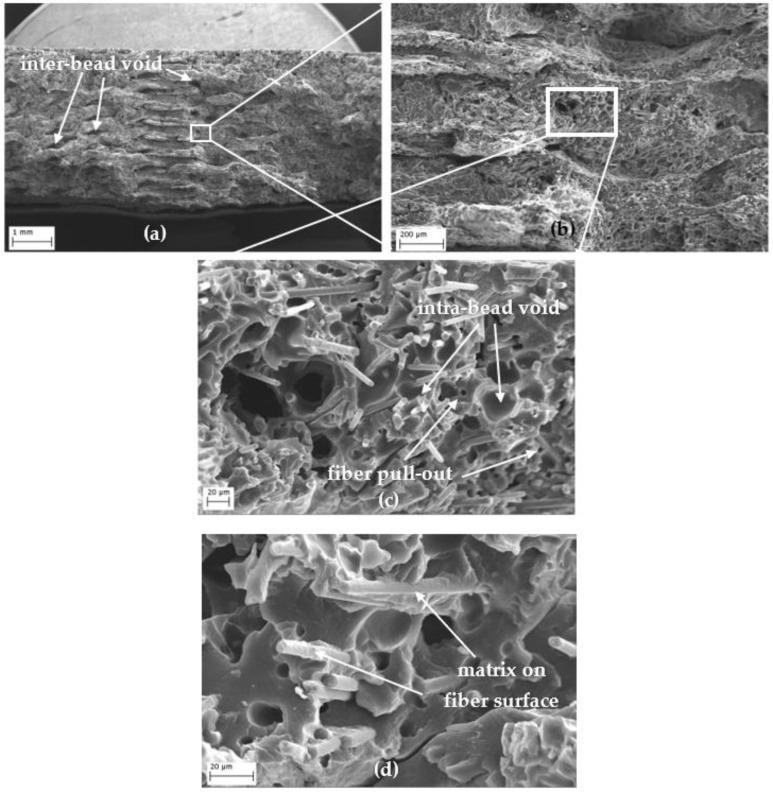
An SEM image of the fracture surface of the CF-reinforced tensile samples showing fiber pull-out, inter, and inter-bead void structures. (**a**–**c**) The zoomed micrograph series and (**d**) the matrix on the fiber surface.

SEM images given in Figure 11 reveal the microstructure of the fracture surface of a CF-reinforced tensile sample. The inter-bead porosity between the adjacent layers are relatively bigger voids, as shown in Figure 11a. These voids occurred because of the lack of complete geometric filling between beads. The intra-bead porosity formed due to flow incompatibility between matrix and carbon fiber and due to pores generated during high-temperature extrusion (Figure 11c). Fiber pull-out also can be seen in (zoomed) images (Figure 11c). The average diameter of the carbon fibers was not specified in the TDS but the measurement from SEM images was around 7 µm. Figure 11d shows the matrix on the fiber surface, proving that the fiber–matrix interface is ideal.

## 4. Conclusions

In this investigation, the effects of fiber reinforcement addition, raster angle, and shell on mechanical properties were evaluated. Additively manufactured tensile, compression, and bending test samples using PA and PA–CF filaments were used. The porosity of manufactured samples was also discussed. The main conclusions can be summarized as follows.

- Carbon fiber reinforcement, regardless of the raster angle, decreased toughness and ductility and increased tensile strength and stiffness. The increase in tensile strength at the 0° samples was obtained as 136%.
- For PA, the obtained elongation at the break value of the ±45° raster angle was 79% and 76% lower than the 0° and 90° angles, respectively. In addition, a decrease of 39% and 24% was observed in the tensile strength, respectively.
- PA and PA–CF samples exhibited almost the same tensile strength values due to the fact that failure takes place due to the weak bonding strength between adjacent rasters.
- The shell layers produced along the loading direction decreased the surface roughness and, because of this reason, the tensile strength of the framed samples was higher than the unframed ones.
- The PA sample showed a compression behavior like a cellular structure due to the naturally inherent structure of the FDM printed sample.The compression rate and printing pattern did not show a significant effect on mechanical properties.
- Similar to the tensile test, the higher flexural strength was observed for the 0° raster angle for both PA and PA–CF.
- A 15% carbon fiber addition increased the porosity from 6.3% to 10.3%.

## Figures and Tables

**Table 1:** Printing parameters.

Parameters	PA	PA–CF
Layer thickness	0.2 mm	
Extrusion width	0.96 mm	
Nozzle diameter	0.8 mm	
Printing speed	30 mm/s	
Nozzle temperature	240 °C	275 °C
Bed Temperature	90 °C	105 °C

**Table 2:** Standards used for mechanical tests and specimen dimensions.

Test Type	ASTM Standard	Length (mm)	Width (mm)	Thickness (mm)	Test Speed (mm/min)	Number of Specimens
Tensile	D638	165	13	4	10	5
Compression	D695	25.4	12.7	12.7	10, 150	3
3-Point Bending	D790	127	12.7	3.2	10	5

**Table 3:** Nomenclature showing filament material, raster orientation, and frame info.

Infill Raster Orientation	PA (Polyamide)	PA–CF (Carbon Fiber Reinforced Polyamide)	PA–CF (Framed with Two Shell Layers)
**0°**	PA0	PA–CF0	-
**45°**	-	PA–CF45	PA–CF45f
**±45°**	PA ± 45	PA–CF ± 45	PA–CF ± 45f
**90°**	PA90	PA–CF90	PA–CF90f

**Table 4:** Tensile properties of PA and PA–CF.

	PA			PA–CF Unframed				PA–CF Framed		
Raster Angle	0°	±45°	90°	0°	45°	±45°	90°	45°	±45°	90°
Tensile Strength (MPa)	40.5	24.6	32.2	95.4	72.7	58.4	31.4	88.0	106.6	88.7
Elongation at Break (%)	19.6	4.1	17.4	1.3	2.6	1.9	1.6	1.9	1.8	1.9
Elastic Modulus (MPa)	1102	849	840	8846	4026	3820	2662	5799	7776	6032
Toughness (J·m^−3^·10^3^)	6988	739	4990	728	1214	694	329	1079	1156	1104

## Data Availability

Not applicable.
